# Investigating the impact of environmental factors on West Nile virus human case prediction in Ontario, Canada

**DOI:** 10.3389/fpubh.2023.1100543

**Published:** 2023-02-17

**Authors:** Laura Albrecht, Kimberly A. Kaufeld

**Affiliations:** ^1^Statistical Sciences Group, Los Alamos National Laboratory, Los Alamos, NM, United States; ^2^Department of Applied Mathematics and Statistics, Colorado School of Mines, Golden, CO, United States

**Keywords:** West Nile virus, zero-inflated Poisson, mosquito, human health, spatiotemporal

## Abstract

West Nile virus is the most common mosquito borne disease in North America and the leading cause of viral encephalitis. West Nile virus is primarily transmitted between birds and mosquitoes while humans are incidental, dead-end hosts. Climate change may increase the risk of human infections as climatic variables have been shown to affect the mosquito life cycle, biting rate, incubation period of the disease in mosquitoes, and bird migration patterns. We develop a zero-inflated Poisson model to investigate how human West Nile virus case counts vary with respect to mosquito abundance and infection rates, bird abundance, and other environmental covariates. We use a Bayesian paradigm to fit our model to data from 2010–2019 in Ontario, Canada. Our results show mosquito infection rate, temperature, precipitation, and crow abundance are positively correlated with human cases while NDVI and robin abundance are negatively correlated with human cases. We find the inclusion of spatial random effects allows for more accurate predictions, particularly in years where cases are higher. Our model is able to accurately predict the magnitude and timing of yearly West Nile virus outbreaks and could be a valuable tool for public health officials to implement prevention strategies to mitigate these outbreaks.

## 1. Introduction

West Nile virus (WNV) is the most prevalent mosquito-borne disease in North America (1). The virus is transmitted from mosquitoes to humans through biting. WNV can range from no symptoms to severe, where the illness affects the central nervous system. Only 20% of people infected with WNV will develop symptoms while <1% of people develop severe neurological symptoms (2, 3). It was first detected in the United States in 1999 and spread quickly over the next 5 years across the US and Canada and has become endemic (4, 5). Since WNV emerged in the United States, it has become the largest arboviral infection in the contiguous United States (6). The first human case of WNV was detected in Ontario, Canada in 2002 (7). Since then, a WNV surveillance program was implemented in Canada to monitor WNV prevalence in humans, mosquitoes, birds, and other mammals (7). WNV transmission occurs mainly through mosquitoes that can become infectious after biting an infected bird (4). Humans and other mammals are dead end hosts that can become infectious but are unable to transmit the virus back to uninfected mosquitoes (2, 4).

Many environmental factors are known to have an effect on WNV transmission as well as on the mosquito life cycle. In a study by García-Carrasco et al. (8), WNV outbreaks were predicted in Europe using an environmental and spatio-environmental risk model. They found that maximum temperatures, presence of rivers, and density of horses and poultry were the best environmental predictors for WNV. In terms of predicting the location of WNV outbreaks, the spatio-environmental model which incorporates latitude and longitude as a proxy for spatial structure predicted the best, suggesting that bird migration rate also plays a role in the geographical pattern of WNV. Another environmental factor that has been correlated with incidence rates for West Nile Virus is the normalized difference vegetation index (NDVI) (9, 10). NDVI provides an index for healthy vegetation and serves as a proxy for suitable conditions for mosquito development (11).

Mosquitoes carry the potential to transmit pathogens from reservoir hosts to humans. In particular, *Culex* mosquitoes are the main vectors involved in the spread of WNV in North America due to their preference for avian blood meals, especially the American Robin (4, 12–14). Giordano et al. (13) found the abundance of bird species can have unpredictable impacts on transmission of the virus due to the feeding preferences of mosquitoes on certain vectors. In late summer, *Culex* sp feeding behavior shifts away from birds to human blood meals (15). This coincides with the peak of human cases in most years.

Climate warming may also change the expansion of vector hosts, leading to WNV spread and become more severe in new parts of the world (16, 17). It can lead to an increase in the frequency and intensity of extreme weather events, such as heatwaves, floods or droughts, which could intensify the interaction between vectors, viruses and hosts (18, 19). Drought, for example, can lead to an increase in the abundance of mosquitoes (20, 21). In regards to Canada, Ludwig et al. (22) showed that as temperatures continue to rise due to climate change, there is an increased risk of vector borne diseases in Canada.

Public health agencies across the US and Canada collect records on mosquito abundance and viral testing for WNV through mosquito trap data as well as human cases (2, 7). Through these programs, the mosquitoes are classified and counted by species based upon geographic locations. As WNV is of great concern, Temple et al. (23) constructed a presence/absence model to account for viral presence in mosquitoes, while accounting for underlying environmental variables for the presence of West Nile virus mosquito presence. They found a higher rate of mosquitoes in the summer, with the leading predictors found to be the number of freezing weeks, urban landscapes and the proportion of *Culex* species. However, this study was tied to mosquitoes instead of human cases. Giordano et al. (13) conducted the first epidemiological study of human WNV cases in Ontario using data from 2002 to 2013 in seven of the most populous PHUs. They found a positive correlations for temperature, cumulative precipitation, mosquito abundance, and the minimum infection rate. This study also found strong spatial autocorrelation of the positive mosquito pools but did not explore any spatial structure in the human WNV case data. We develop a statistical model for human WNV cases to better understand the relationship between human cases and environmental variables across the 27 PHUs in lower Ontario. We use a zero-inflated Poisson model, which is used to model data that has an excess of zeros. We compare a spatial and non-spatial version of the model to assess if there is an underlying spatial structure to the WNV counts. To our knowledge, this has not been done before.

The paper is presented as follows. In Section 2, we introduce the WNV data in Ontario, Canada and describe the environmental variables used in the analysis. We also present the spatial and non-spatial zero-inflated Poisson model for modeling WNV human cases. In Section 3 we present and compare model results for WNV. We conclude with a short discussion on the zero-inflated Poisson model and future use in human case disease modeling.

## 2. Materials and methods

### 2.1. Data sources

**Figure 1:**
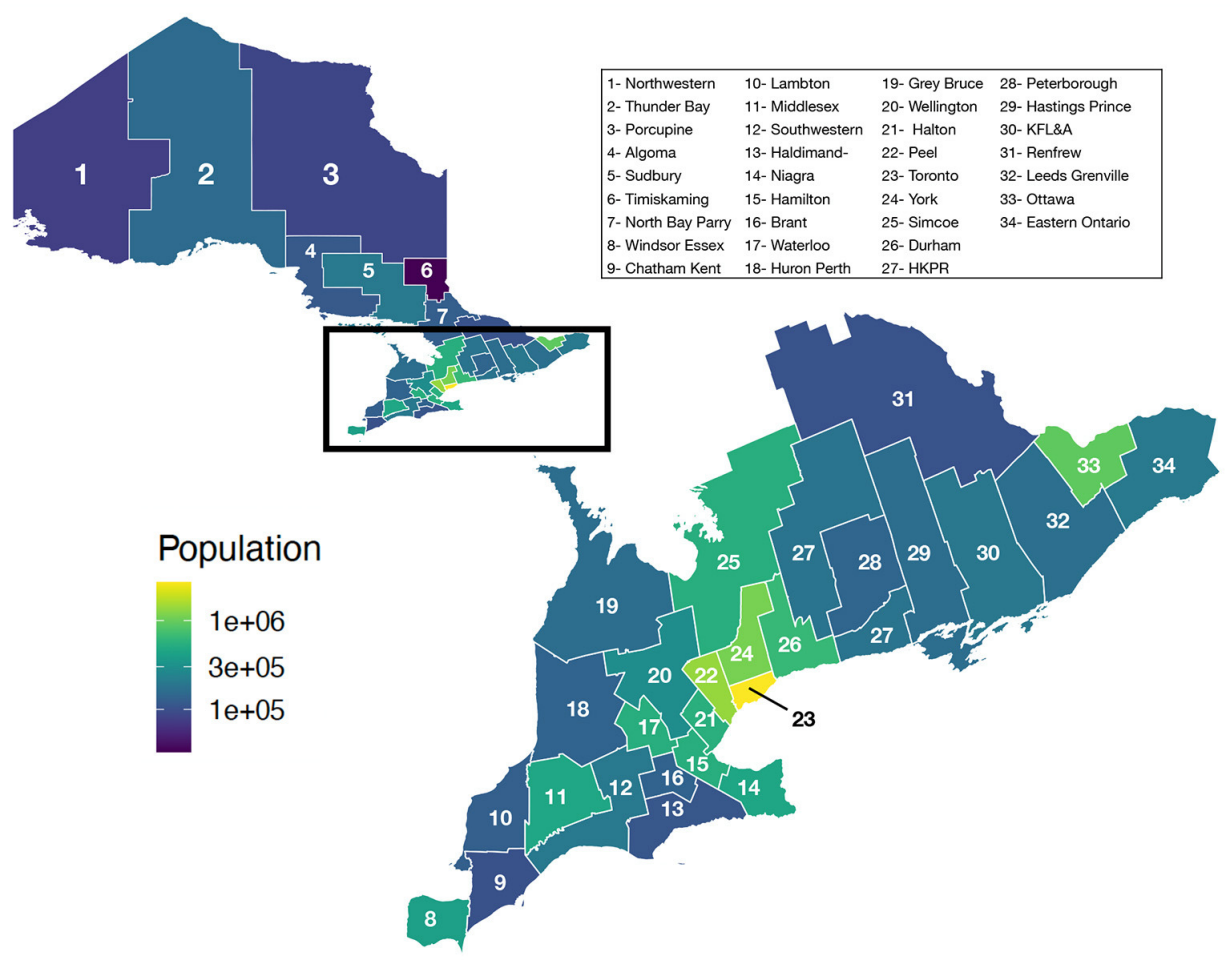
Population of Ontario by PHU.

**Figure 2:**
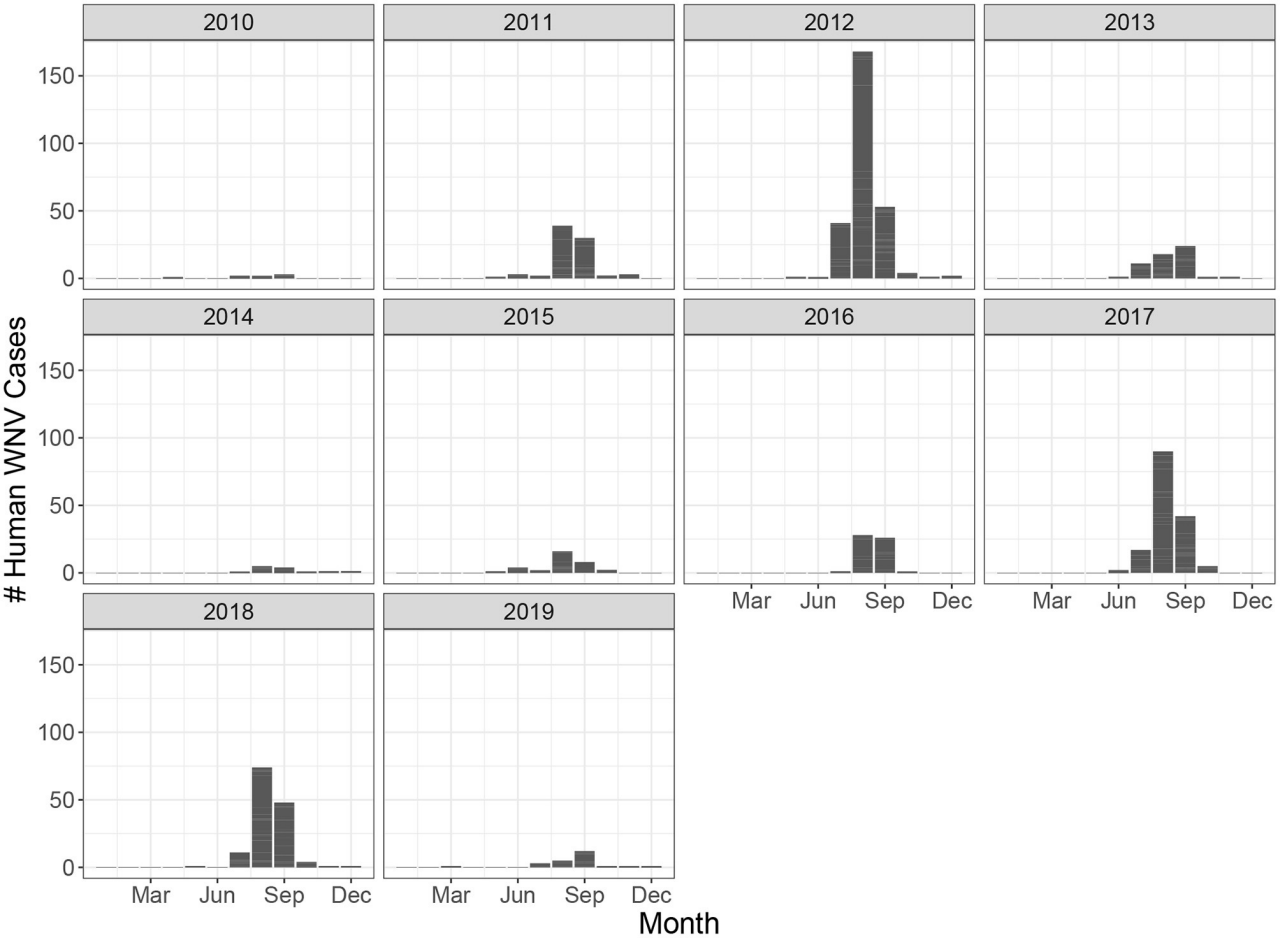
Monthly human West Nile virus cases in all of Ontario from 2010 to 2019.

**Figure 3:**
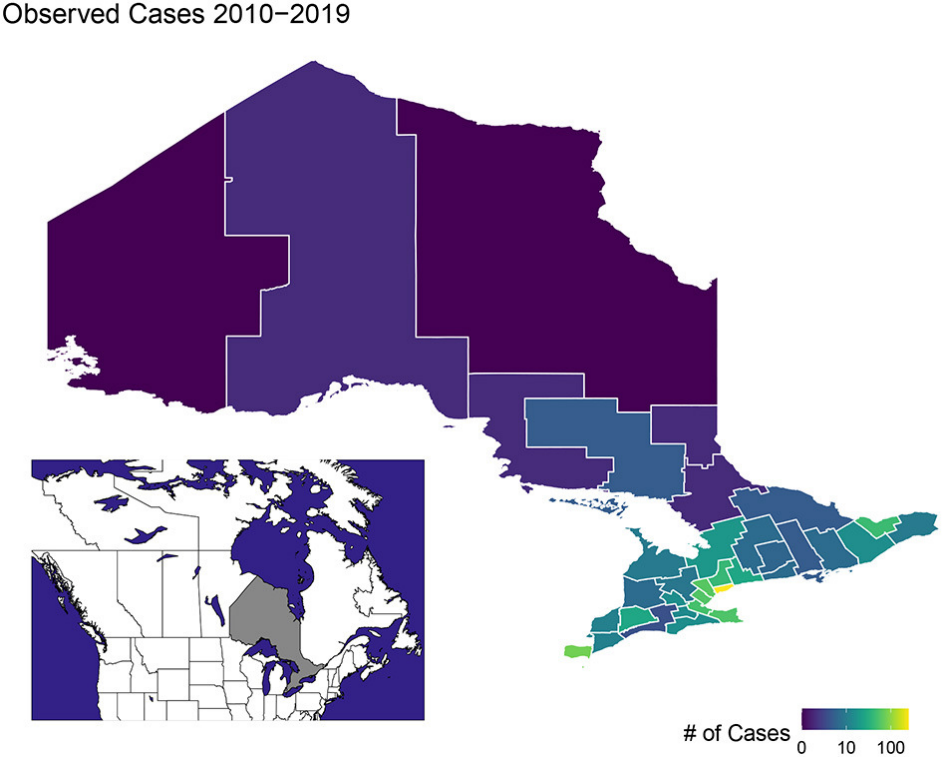
Map of total human West Nile virus cases in Ontario from 2010 to 2019.

Ontario is the most populous province in Canada with just over 15 million residents and the third largest in size spanning 1.076 million km^2^ (Figure 1). Ontario is divided into 34 Public Health Units (PHUs). We collected monthly human case data from 2010 to 2019 for each PHU from the Public Health Ontario website (24). We focus our analysis on the 27 PHUs located in lower Ontario as upper Ontario is sparsely populated and has had only 8 total cases reported across all 10 years in our data set. Most human cases are reported between May and October with a peak in August while very few cases are observed in the winter months (Figure 2). We observe high variability in human cases from year to year with some years having very few cases, for example 2010 and 2014, and others having large outbreaks i.e., 2012, 2017. It appears some spatial correlation may be present in the case data as the majority of cases are clustered around Toronto (Figure 3). While severe cases are likely to be detected, most cases will not be reported. Hence, the WNV cases reported are only a small subset of the true number of infections.

We collected mosquito trap data, bird abundance, temperature and precipitation, land cover, population, and Normalized Difference Vegetation Index (NDVI) data to be used as potential covariates to model the human WNV cases.

Since 2002, each PHU has used mosquito traps to obtain information on mosquito abundance and viral testing. Officials place light traps to attract mosquitoes and return to collect the traps on a weekly or biweekly basis from May through October. They record the total abundance of mosquitoes collected in the trap. A subset of this total is then further identified by species. Then, mosquitoes are pooled into groups and tested for WNV in a lab. Each pooled sample is recorded as either positive or negative. A positive pool indicates that at least one mosquito in the pool is positive but does not provide insight into the number or fraction of mosquitoes in the pool that are positive. Pool sizes range from 1-60 with the majority of pools containing less than 10 mosquitoes. To account for the differences in pool sizes, the mosquito infection rate is often used as a proxy.

A common correction is to use the Minimum Infection Rate (MIR) which assumes that only one individual is positive for every positive pool. This effectively ignores the pooling information and provides a lower bound on the infection rate. This method is appropriate only when infections are considered to be low (25). Otherwise, the rate tends to be too narrow as it does not reflect the information lost in pooling. Instead, since our data contains varying pool sizes, we use the MLE method as described in Biggerstaff (26) which incorporates the different pool sizes in the infection rate calculation. Using this method, we assume the number of positive mosquito pools, *X*_*i*_ for *i* = 1, ..., *M*, where M is the number of distinct pool sizes, is binomially distributed as shown in Equation 1 where *n*_*i*_ is the number of pools of size *m*_*i*_, and *X*_*i*_ is the number of *n*_*i*_ pools observed that are positive.


(1) $$\begin{array}{l} X_{i}\sim Binomial\left(n_{i},1-{\left(1-p\right)}^{m_{i}}\right) \end{array}$$

We gathered observational bird data from Cornell's citizen science eBird project (27). We calculated the relative abundance for several bird species known to be good carries of WNV including the American Robin, House Sparrow, and American Crow (28). Relative abundance is used to correct for the inherent sampling bias present in the eBird data. Kilpatrick (29) found abundance of bird species can have unpredictable impacts on transmission of the virus due to the feeding preferences of mosquitoes on certain hosts. Feeding preferences of *Culex* mosquitoes have also been observed to change throughout the season with mosquitoes preferring human blood meals more later in the year (12).

We collected temperature and precipitation data from weather stations in Ontario using the weathercan package in R (30, 31). We obtained 12 land classification proportions that had been inferred from satellite imagery (32). Census data was collected from Statistics Canada and includes population, population density, number of dwellings, dwell density, and area (33). We collected monthly NDVI data at 1km spatial resolution from the MODISstp package (34). All covariates were aggregated to obtain monthly averages across each PHU.

### 2.2. Model

The West Nile Virus case data contains many months in which zero cases are reported in a given PHU. Due to the excess of zeros, we use Bayesian zero-inflated Poisson (ZIP) model. The ZIP model allows us to model zero observations from two distinct processes, the one associated with the number of cases, and a model for the excess zeros. The model has two parts, a Poisson count model and a logit model for the zeros. We fit two different ZIP models, a spatial and non-spatial model. We will describe the non-spatial version and extrapolate to the spatial version.

For public health unit *i*, (*i* = 1, ..., 27), and month *t, t* = (1, ..., 120), define *Y*_*it*_ to be the number of human West Nile Virus cases where cases start in January 2010. We model the West Nile cases as a Poisson generalized linear model,


(2) $$\begin{array}{l} Y_{it}|X_{it}\sim Poisson\left(E_{it}e^{x_{it}^{T}\beta }\right) \end{array}$$

Where *E*_*it*_ is the expected number of disease counts in the absence of covariate effects. This is the number of cases expected in each PHU if each person is equally likely to get West Nile virus. However, when there are no cases observed in PHU *i* and time *t* we assume that these zeroes occur as structural zeroes with probability ω_*it*_, and model the zero case counts as Poisson variables with probability (1−ω_*it*_). We assume the following Bayesian zero-inflated Poisson model:


(3) $$P(Y_{it}=y_{it}|\omega_{it},\lambda_{it})=\left\{ \begin{array}{l} \omega_{it}+(1-\omega_{it})\text{exp}(-\lambda_{it})\text{ for }y_{it}=0\\ (1-\omega_{it})\text{exp}(-\lambda_{it})\lambda_{it}^{y_{it}}/y_{it}!\text{ for }y_{it}>0 \end{array}\right.$$

We assume a logit link for each probability, ω_*it*_, and relate these quantities to environmental variables, temporal and, in the spatial version, spatial random effects. More specifically, we assume a Poisson distribution of the cases as


(4) $$\begin{array}{l} \text{log}\left(\lambda_{it}\right)=X_{it}\beta +\phi y_{i,t-1} \end{array}$$

Where ***X***_*it*_ is a vector of covariates related to exposure risk, **β** is the vector of regression coefficients. We include a temporal random effect, ϕ, which is an autoregressive process. It is used to account for the idea that the cases within PHU *i* during month *t* are closely related to the cases the month before, i.e., PHU *i* at month *t*−1. We assume that the number of cases relate to mosquito infection rate, bird abundance, NDVI, temperature, and precipitation. The model for the zero cases also includes a random effect to account for temporal autocorrelation as in the Poisson model. That is,


(5) $$\begin{array}{l} \text{logit}\left(\omega_{it}\right)=Z_{it}\gamma +\phi y_{i,t-1} \end{array}$$

Where ***Z***_*it*_ is a vector of covariates related to the structural zeros, **γ** is the vector of regression coefficients.

The spatial version of the zero-inflated Poisson model borrows information from neighboring sites. For example, for one public health unit, *i*, if the neighboring public health units have cases, it is more likely that the public health unit, *i*, will also have WNV cases. The spatial model is modified slightly from Equation (4), as a spatial random effect, *u*_*i*_ is incorporated for each PHU *i*. That is,


(6) $$\begin{array}{l} \text{log}\left(\lambda_{it}\right)=X_{it}\beta +u_{i}+\phi y_{i,t-1} \end{array}$$

(7) $$\begin{array}{l} \text{logit}\left(\omega_{it}\right)=Z_{it}\gamma +\phi y_{i,t-1} \end{array}$$

Similarly to Equation (4), ***X***_*it*_ and ***Z***_*it*_ are vectors of covariates associated with the non-zero and zero process respectively. The **β** and **γ** are vectors of coefficients associated with these covariates and *u*_*i*_ are the spatially correlated random effects for the Poisson process.

We utilize a Bayesian paradigm and put multivariate normal priors on the regression coefficients. We put an intrinsic conditional autoregressive prior on *u*_*i*_ (see Supplementary material for details). Posterior estimates are found using a random walk Metropolis-Hastings Markov Chain Monte Carlo (MCMC) in NIMBLE (35–37). The MCMC simulation was run for three independent chains for 500,000 iterations with a burn-in period of 100,000 iterations and a thinning level of 100. Posterior trace plots were inspected to ensure convergence of each chain.

For model selection, we utilized the Watanabe-Akaike information criteria (WAIC) as a measure of out-of-sample predictive accuracy which adds a correction for the effective number of parameters to adjust for overfitting (38, 39). A lower WAIC values indicates a better model fit. NIMBLE calculates the WAIC from the posterior samples produced by the MCMC algorithm. All temporal variables were lagged by 0, 1, and 2 months and the WAIC values of each model were compared to select the most appropriate lags.

In order to test the predictability of our model, we held out data from 2018 to 2019 for posterior prediction. We drew 1,000 samples from the posterior distributions of each parameter to obtain 1,000 predictions of the number of cases in each month and PHU ($Y_{it}^{pred}$). To evaluate our predictions, we calculate the mean squared error between the predicted number of cases and the true number of cases as in Equation 8.


(8) $$\begin{array}{l} \text{MSE}=\frac{1}{n}\underset{i,t}{\sum }{\left(Y_{it}^{pred}-Y_{it}\right)}^{2} \end{array}$$

We also compare visually the median number of predicted cases in each year to the observed data.

## 3. Results

The temporal and spatiotemporal models were compared to see if the spatial dependence from the neighboring sites adds more value for predicting WNV cases. As part of the modeling, we explored different lagged windows, i.e., 1 or 2 month temperature or precipitation lag. This was based on the knowledge that it takes a month or two for a WNV cases to be reported (40).

**Figure 4:**
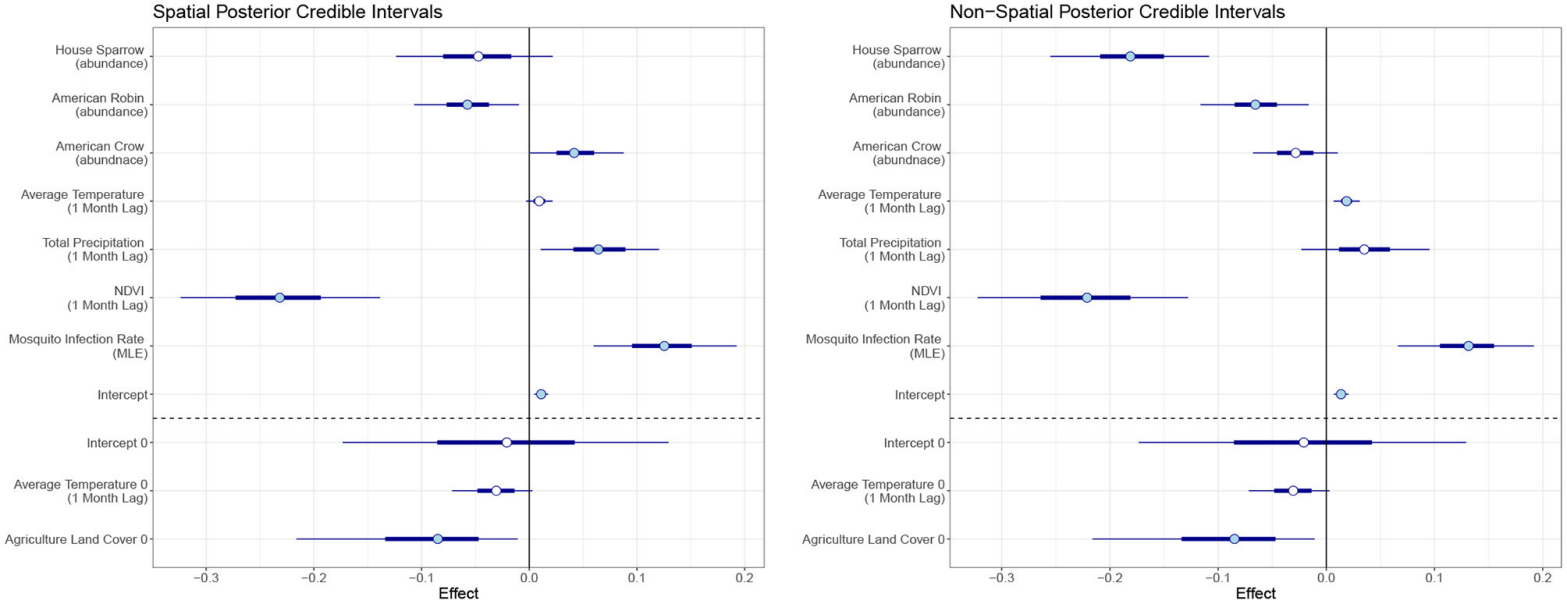
Posterior credible intervals of the effect of each covariate for the spatiotemporal and temporal model. The skinny outer line indicates the 95% credible interval, the thick line is the 50% credible interval, and the circlular point is the median. A filled circle indicates the covariate is significant at the 95% level.

In our final model, we used mosquito infection rate MLE, NDVI (1 month lag), average temperature (1 month lag), total precipitation (1 month lag), and crow, robin, and sparrow abundance for the Poisson covariates. For the structural zero covariates, we used percent of agriculture and average temperature lagged by 1 month. Posterior effect estimates for the spatial and non-spatial model are shown in Figure 4. A filled circle indicates the predictor is significant at the 95% level. The thicker line shows the 50% credible interval and the thin line shows the 95% credible interval. Point estimates and credible interval values are also included in Table 1.

**Table 1:** Posterior results for the spatial and non-spatial model.

		**Spatial**			**Non-Spatial**		
	**Parameter**	**Mean**	**SD**	**95% CI**	**Mean**	**SD**	**95% CI**
Poisson	Average Temperature (°C)	0.009	0.008	(–0.006, 0.024)	0.019	0.007	**(0.005, 0.034)**
	Total Precipitation (cm)	0.065	0.034	**(0.011, 0.121)**	0.035	0.035	(–0.033, 0.103)
	Mosquito infection rate (MLE)	0.125	0.042	**(0.043, 0.206)**	0.130	0.038	**(0.052, 0.204)**
	NDVI	–0.231	0.058	**(–0.341, –0.122)**	–0.022	0.006	**(–0.034, –0.011)**
	American robin	–0.058	0.029	**(–0.119, –0.001)**	–0.065	0.029	**(–0.125, –0.01)**
	House sparrow	–0.049	0.045	(–0.137, 0.033)	–0.181	0.045	**(–0.269, –0.096)**
	American crow	0.043	0.026	**(0.001, 0.097)**	–0.029	0.024	(–0.076, 0.016)
	Intercept	0.011	0.004	**(0.003, 0.019)**	0.014	0.004	**(0.006, 0.022)**
Zero- Inflated	Agriculture land cover	–0.096	63.731	**(–0.238, –0.006)**	–0.097	0.063	**(–0.241, –0.005)**
	Average temperature (°C)	–0.032	23.100	(–0.077, 0.006)	–0.031	0.024	(–0.081, 0.006)
	Intercept	–0.022	94.348	(–0.206, 0.16)	–0.014	0.097	(–0.213, 0.17)

This shows the mean, standard deviation, and 95% Credible Interval (CI) of the effect size of each parameter from the posterior draws. Credible intervals in bold indicate the parameter is significant at the 95% level.

In the temporal model, we found MLE and average temperature had a significant positive effect on human WNV cases at the 95% level with mosquito MLE having the largest effect. NDVI, sparrow and robin abundance had negative effects. Agriculture land cover showed a significant negative effect on the log-odds for the structural zeroes. That is, an increase in the percent of land cover that is agricultural leads to a reduction in the log-odds for structural zeros.

For the spatiotemporal model, total precipitation and crow abundance also have a significant positive effect at the 95% level. All other estimates are similar though the magnitudes differ slightly from the temporal model. The WAIC values for the non-spatial and spatial models are 1,404 and 1,328, respectively, which indicates the spatial model performs slightly better than the non-spatial version.

**Figure 5:**
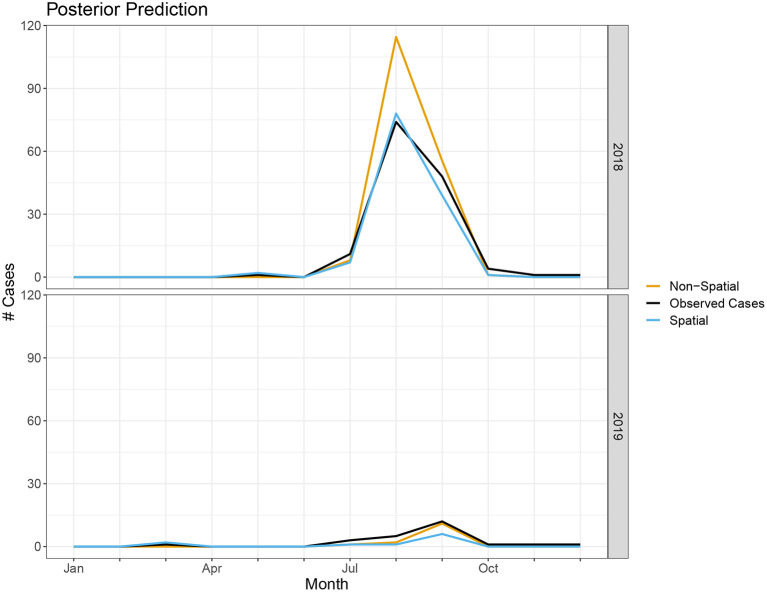
Number of cases predicted in spatial and non-spatial model compared to true observed cases in 2018 and 2019.

**Figure 6:**
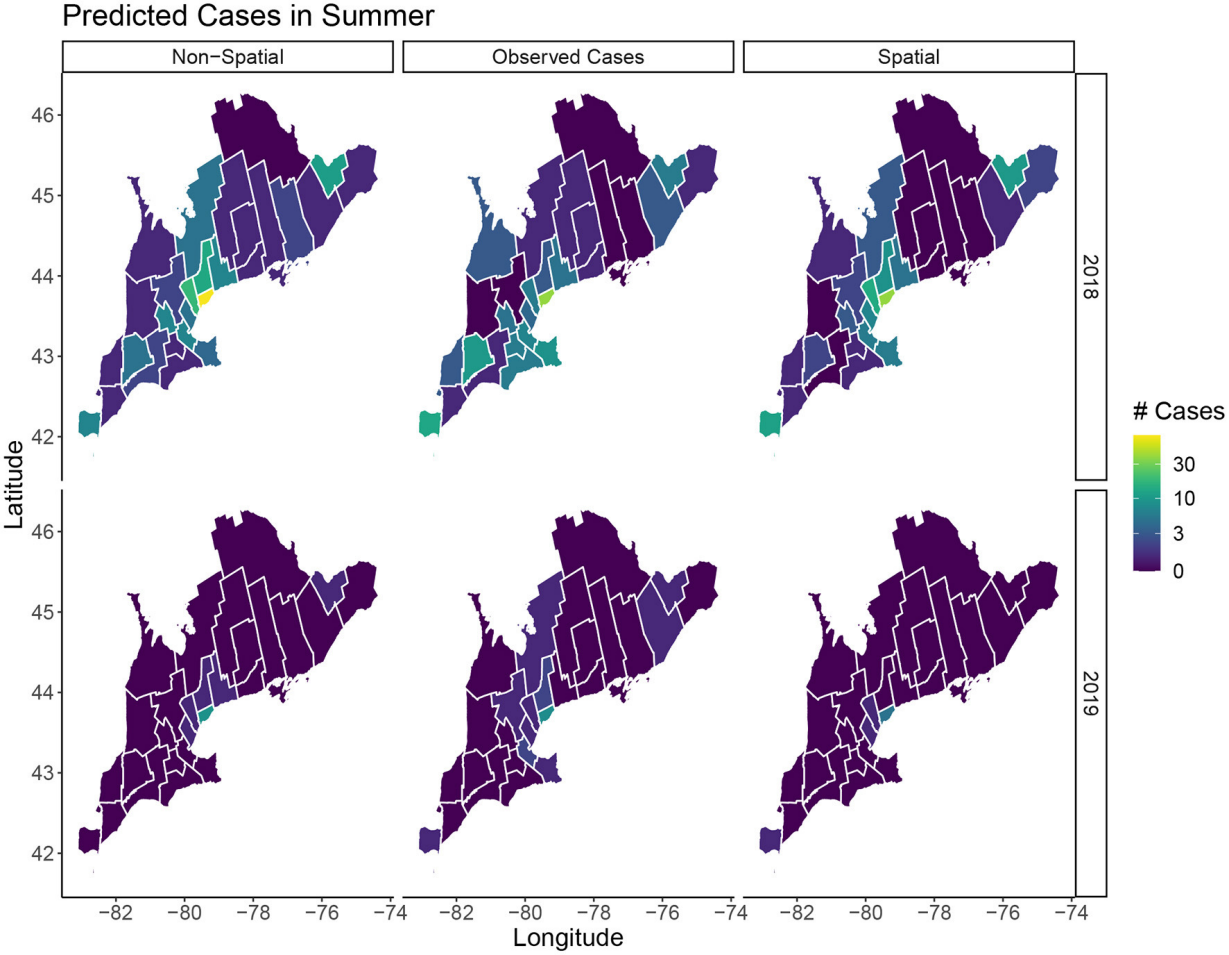
Predicted cases in July, August, and September for 2018 and 2019. The left column shows the median predicted number of cases in the non-spatial model, the center column is the true observed cases, and the right column shows the median predicated cases in the spatial model.

In terms of posterior predictions, the spatiotemporal model also performs slightly better with an MSE of 1.17 compared to the temporal model MSE of 2.02. Median predictions for the spatial and non-spatial model are shown in Figure 5. We see the spatial model more accurately captures the peak infections in the summer of 2018 when cases are highest. Maps of the median posterior predictions by PHU for the summer months (July, August, and September) are shown in Figure 6. The non-spatial model in 2018 highly over predicts the number of cases in the greater Toronto region while the spatial model predictions are close to the observed data. Very few cases were observed in 2019 and both model predictions are similar to the observed data. We calculated the difference between the number of cases observed in each month with the median number of cases predicted. Spatial maps of the differences for summer months in 2018 and 2019 are shown in Supplementary Figure S1. The number of cases predicted in all other seasons are very low and only contain small deviations from the observed data.

## 4. Discussion

Human case data of WNV is complicated to study and has many limitations. Case data is only reported at the monthly timescale while many of our covariates are measured at hourly, daily, or weekly increments. Aggregating these covariates to the monthly scale may lead to a loss of information and may mask the underlying relationship between these covariates and human cases. Additionally, cases are greatly under reported due to a high number of asymptomatic cases and limited surveillance testing. We develop a zero-inflated Poisson model with lagged temporal covariates to help elucidate the relationship between human cases of WNV and environmental variables.

We find the mosquito infection rate correlates strongly with human WNV cases. Average temperature and total precipitation lagged by 1 month also have slight positive correlations with human cases. Higher temperatures result in an increasing abundance of mosquitoes, a shorter extrinsic incubation period, and increased biting rates, thus, leading to a higher probability of transmission of WNV (41). Our results show a seemingly counter-intuitive negative correlation between NDVI and cases since an increase in NDVI should lead to an increase in mosquitoes (42, 43). However, this relationship was previously observed in several other studies of WNV (20, 44). This may be related to the tendency of *Culex* mosquitoes to thrive better in artifical pools of water where fewer predators are present (45). Low NDVI can also be indicative of a drought which may cause mosquitoes and birds to congregate near the remaining bodies of water thus increasing transmission (21). We decided to only include the percent of agriculture among the land cover variables due to the high correlation between the different land cover variables. Miramontes et al. (46) found an association between agricultural activity and human incidence of West Nile virus.

The relationship between birds and WNV is complex due to the shifting seasonal biting preferences of mosquitoes. While robins are known to be good carriers of WNV, we find an increase in the abundance of robins is associated with a decrease in human cases. This could be due to the feeding preference of mosquitoes being skewed toward birds at the times where the abundance of this species is higher. While using relative abundance helps standardize the eBird observations, it is still impacted by factors such as bias in the reporting rate of rare and common species (47).

Our model is able to accurately capture the trends and peak timing of WNV outbreaks. While the posterior estimates for the non-spatial and spatial models are similar, the WAIC values indicate a modest improvement when using the spatial model. Our posterior prediction for 2018 shows the spatial model more accurately predicts the peak number of cases in the summer (Figure 5). The number of cases in 2019 is much lower and the difference between the two model predictions is negligible. The improvements with the spatiotemporal model are modest but, especially when cases are high, it does appear there is a spatial structure to the human cases and including spatial random effects leads to better predictions. Our model's ability to accurately predict the magnitude and timing of yearly WNV outbreaks could be valuable to public health officials to implement prevention strategies and public health announcements to mitigate these outbreaks.

One limitation of this study is the narrow geographic scope. Currently our results are specific to the province of Ontario and cannot be generalized to other locations. Future work applying this model to locations across North America is necessary to determine the reliability of our results in other locations. Expanding this analysis is complicated due to varying levels of data availability in different public health agencies. In the United States, for instance, county level data for human cases is reported on a yearly temporal scale and monthly case data is only available at the state level. Investigating seasonal trends will be more challenging at these larger spatial and temporal scales.

The correlations observed between these environmental factors with human cases indicates climate change could lead to an increase of WNV cases across Ontario. Currently PHUs in upper Ontario have reported very few cases of WNV but increasing temperatures and an increase in drought conditions may lead to more cases in this region in the coming years. Also, an increase in temperature could lead to changes in bird migration patterns and locations. Some species of birds have reportedly been migrating earlier in the spring as a result of warmer spring mean temperatures (48). This could impact the peak timing and length of the WNV disease season. The accurate predictive power of our model could allow us to simulate WNV outbreaks under different climate change scenarios. Knowledge of the intricate relationships between these variables and human cases is vital for planning and prevention of future outbreaks of WNV.

## Data availability statement

Publicly available datasets were analyzed in this study. This data can be found at: https://www.publichealthontario.ca/en/data-and-analysis/infectious-disease/west-nile-virus.

## Author contributions

LA and KAK: conceptualization, methodology, statistical analysis, and writing. LA: computer programming. All authors contributed to the article and approved the submitted version.
