# Supplementary material for: Investigating the impact of environmental factors on West Nile virus human case prediction in Ontario, Canada
Source: Front Public Health. 2023 Feb 17;11:1100543. doi: 10.3389/fpubh.2023.1100543 (PMC9981635; doi:10.3389/fpubh.2023.1100543)
Supplement: Supplementary file 1 [file Data_Sheet_1.PDF]

Supplementary Material

1 MODEL PRIORS

We assume all $\boldsymbol{\beta}$ and ϕ parameters are independent with $\mathcal{N}(0, 10^2)$ priors. For the u_i parameters, we use the dimension reduction method as developed by Hughes and Haran (2013). Using this formulation speeds up computation and alleviates spatial confounding which leads to better convergence of our MCMC chains.

We set up our spatial random effects as follows. Let $\boldsymbol{A}$ be a 27x27 adjacency matrix of the PHUs with entries given by diag($\boldsymbol{A}$) = $\mathbf{0}$ and $\boldsymbol{A_{ij}} = 1$ if i and j are adjacent and 0 otherwise. Let $\boldsymbol{M}$ be a $27 \times q$ matrix of the first q columns of the Moran basis $\boldsymbol{P}^{\perp}\boldsymbol{A}\boldsymbol{P}^{\perp}$ where $\boldsymbol{P}^{\perp}$ is the projection onto the orthogonal column space of $\boldsymbol{X}$, the $27 \times P$ matrix of time-constant covariates. That is, $\boldsymbol{P}^{\perp} = \boldsymbol{I} - \boldsymbol{X}(\boldsymbol{X'X})^{-1}\boldsymbol{X'}$. We set $u_i = \boldsymbol{M}\eta$ where η is a $q \times 1$ vector of coefficients. We use a prior of η derived from the intrinstic conditional autoregressive (ICAR) model as described in Hughes and Haran (2013) as shown in Equation S1.

$$\eta|\tau \propto \tau^{q/2}\text{exp}(-\frac{\tau}{2}\eta'\boldsymbol{Q_s}\eta) \tag{S1}$$

Here, τ is a smoothing parameter and $\boldsymbol{Q_s} = \boldsymbol{M'QM}$ where $\boldsymbol{Q} = diag(\boldsymbol{A1} - \boldsymbol{A})$ and $\mathbf{1}$ is a vector of 1s. We set $q = 10$, thus reducing the dimension of our spatial effects from 27 to 10.

2 SUPPLEMENTARY TABLES AND FIGURES

2.1 Figures

REFERENCES

Hughes, J. and Haran, M. (2013). Dimension reduction and alleviation of confounding for spatial generalized linear mixed models. *Journal of the Royal Statistical Society: Series B (Statistical Methodology)* 75, 139–159

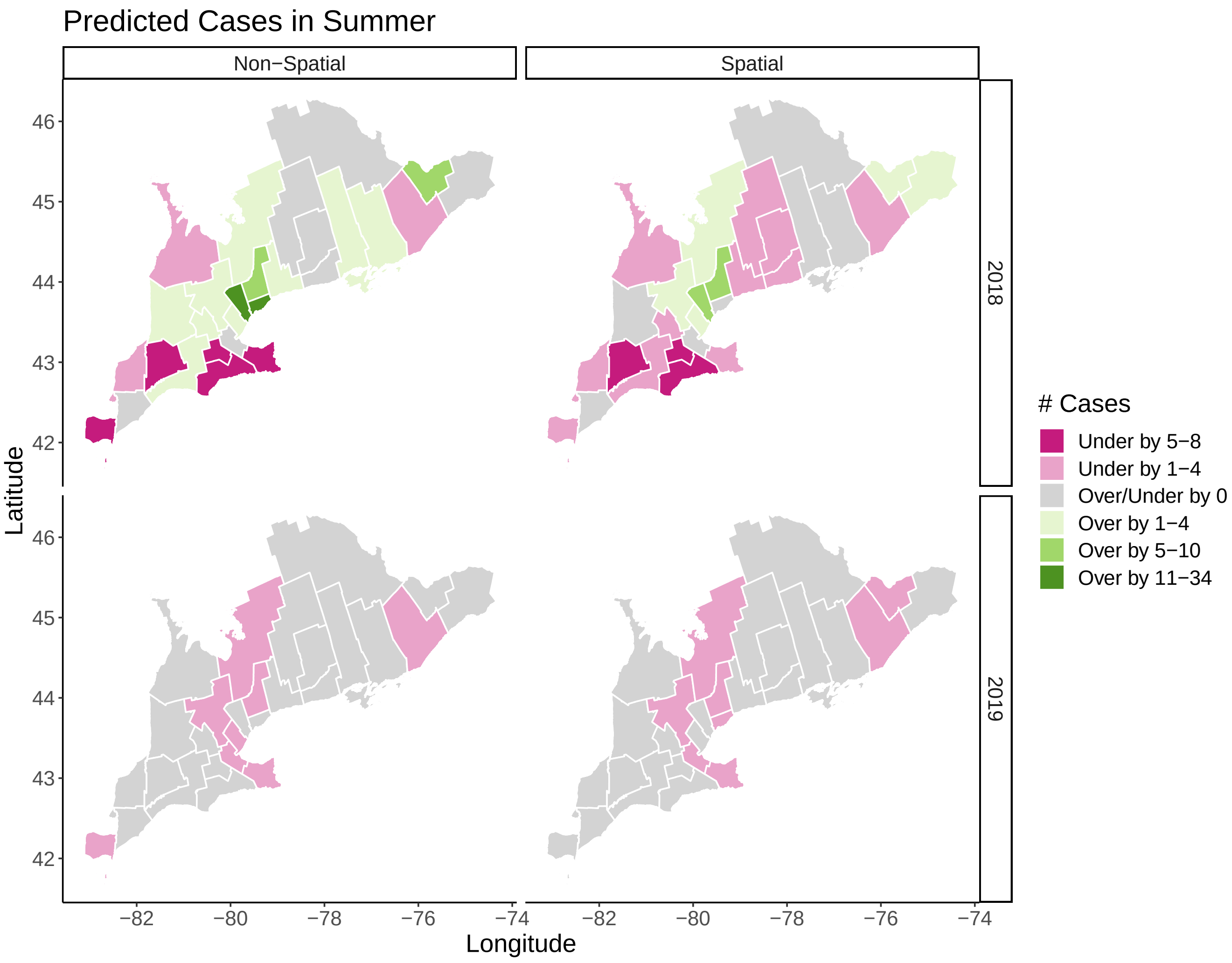

Figure S1. Difference between the observed cases from the median predicted non-spatial (left) and median predicted spatial (right) cases in July-September. The non-spatial model leads to large over predictions in the greater Toronto region in 2018 while the spatial model predictions are much more accurate. The total number of cases is much lower in 2019 and both models predict within 4 cases of the observed data in all PHUs.
